# TRIF signaling is required for caspase-11-dependent immune responses and lethality in sepsis

**DOI:** 10.1186/s10020-018-0065-y

**Published:** 2018-12-27

**Authors:** Yiting Tang, Rui Zhang, Qianqian Xue, Ran Meng, Xiangyu Wang, Yanliang Yang, Lingli Xie, Xianzhong Xiao, Timothy R. Billiar, Ben Lu

**Affiliations:** 1 Department of Physiology, School of Basic Medical Science, Central South University, Changsha, Hunan Province 410000 People’s Republic of China; 2 Department of Hematology and Key Laboratory of Non-resolving Inflammation and Cancer of Hunan Province, The 3rd Xiangya Hospital, Central South University, Changsha, 410000 People’s Republic of China; 3 State Key Laboratory of Medical Genetics, School of Biological Science and Technology, Central South University, Changsha, Hunan Province 410000 People’s Republic of China; 4 Department of Pathophysiology, School of Basic Medical Science, Jinan University, Guangzhou, Guangdong Province 510632 People’s Republic of China; 5 Department of Pathophysiology, School of Basic Medical Science, Central South University, Changsha, Hunan Province 410000 People’s Republic of China; 6 Department of Surgery, University of Pittsburgh Medical Center, Pittsburgh, PA 15213 USA

**Keywords:** Non-canonical inflammasome, Caspase-11, Innate immunity, Sepsis

## Abstract

**Background:**

Caspase-11, a cytosolic receptor of bacterial endotoxin (lipopolysaccharide: LPS), mediates immune responses and lethality in endotoxemia and experimental sepsis. However, the upstream pathways that regulate caspase-11 activation in endotoxemia and sepsis are not fully understood. The aim of this study is to test whether TIR-domain-containing adapter-inducing interferon-β (TRIF) signaling is critical for caspase-11-dependent immune responses and lethality in endotoxemia.

**Methods:**

Mice of indicated genotypes were subjected to endotoxemia or cecum ligation and puncture (CLP) and monitored daily by signs of a moribund state for lethality. Serum interleukin (IL)-1α, IL-1β, IL-6 and tumor necrosis factor (TNF) were measured by ELISA. Data were analyzed by using student’s t-test or one-way ANOVA followed by post-hoc Bonferroni test. Survival data were analyzed by using the log-rank test.

**Results:**

Blockade of type 1 interferon signaling or genetic deletion of TRIF or guanylate-binding proteins (GBPs) prevented caspase-11-dependent immune responses, organ injury and lethality in endotoxemia and experimental sepsis. In vitro, deletion of GBPs blocked cytosolic LPS-induced caspase-11 activation in mouse macrophages.

**Conclusions:**

These findings demonstrate that TRIF signaling is required for caspase-11-dependent immune responses and lethality in endotoxemia and sepsis, and provide novel mechanistic insights into how LPS induces caspase-11 activation during bacterial infection.

## Background

Increased levels of circulating LPS are encountered in sepsis and removal of LPS is beneficial to septic patients (Angus and van der Poll 2013; Ronco et al. 2010). Endotoxemia-induced lung injury and lethality depends on the activation of caspase-11, an intracellular LPS receptor that triggers a lytic form of cell death, termed pyroptosis (Kayagaki et al. 2011; Hagar et al. 2013; Kayagaki et al. 2013; Wang et al. 1998; Cheng et al. 2017). In this context, activated caspase-11 cleaves gasdermin D (GSDMD) into pore-forming peptides that disrupt the cell membranes (Kayagaki et al. 2015; Ding et al. 2016). This process leads to the release of alarmins, such as interleukin (IL)-1α, and non-canonical activation of the NLR pyrin domain-containing protein 3 (NLRP3) inflammasome, an intracellular protein complex that mediates the maturation of IL-1β through caspase-1 (Kayagaki et al. 2011; Kayagaki et al. 2015). Genetic deletion of Caspase-11 or GSDMD confers significant protection against lethal endotoxemia (Kayagaki et al. 2011; Hagar et al. 2013; Kayagaki et al. 2013; Wang et al. 1998; Cheng et al. 2017; Kayagaki et al. 2015). Pharmacological inhibition of caspase-11 by oxidized phospholipid (oxPAPC) or stearoyl lysophosphatidylcholine significantly promotes survival in endotoxemia (Chu et al. 2018; Li et al. 2018). However, the upstream pathways that regulate caspase-11 activation in endotoxemia are not fully understood.

Recent studies show that bacterial outer membrane vesicles, membrane-enclosed entities released by variety of bacteria, could efficiently delivers LPS into the cytosol and subsequently leads to activation of caspase-11 (Meunier et al. 2014; Finethy et al. 2017). We and others further demonstrate that the TIR-domain-containing adapter-inducing interferon-β (TRIF) signaling is critical for OMVs-induced caspase-11 activation (Santos et al. 2018; Gu et al. 2018). In this scenario, TRIF signaling mediates the production of type 1 interferon, which in turn induces the expression of guanylate-binding proteins (GBPs). The latter is required for OMVs- or Gram-negative bacteria-induced caspase-11 activation (Santos et al. 2018; Gu et al. 2018). These observations prompt us to test whether TRIF signaling is critical for caspase-11-dependent immune responses and lethality in endotoxemia.

## Methods

### Mice

Male wild-type (WT) C57BL/6 mice, B6.129S4 (D2)-Casp4tm1Yuan/J (Caspase-11 KO) mice, C57/B6 trif-LPS2 (TRIF KO) mice and B6.129S2-Ifnar1tm1Agt/Mmjax (IFNaβR KO) mice were purchased from the Jackson Laboratory. GBPchr3 KO mice and GBP2 KO mice were generated as described previously (Yamamoto et al. 2012; Degrandi et al. 2013). Receptor interacting protein 3 (Rip3) KO mice were generated by the transcription activator-like effector nucleases (TALENs)-mediated gene-disruption method in a C57BL/6 background, as described previously (Wu et al. 2013).

Mice were bred in the animal facilities of Central South University. Experimental protocols were approved by the Institutional Animal Care and Use Committees of Central South University.

### Reagents

Ultrapure LPS (*E. coli 0111:B4*) for in vitro experiments were obtained from InvivoGen. LPS (*E. coli 0111:B4*) for endotoxemia experiments were obtained from Sigma.

### Endotoxemia model

Male or female mice that were 25 to 30 g in weight were injected intraperitoneally with 10 mg/kg LPS (*E. coli 0111:B4*, Sigma). Serum samples were collected at 16 h after LPS injection for the detection of IL-1α, IL-1β, TNF-α and IL-6. Mice injected intraperitoneally with 10 mg/kg LPS were sacrificed 8 h later to measure serum alanine aminotransferase (ALT), creatinine (Cre) levels. Lung specimens were stained with H&E.

For survival experiments, mice were injected intraperitoneally with 40 mg/kg LPS and monitored daily by signs of a moribund state for lethality.

### CLP procedure

Experimental sepsis was induced by cecal ligation and puncture (CLP). Male or female mice that were 25 to 30 g in weight were used. The skin was disinfected with a 2% iodine tincture. Laparotomy was performed under 2% isoflurance (Piramal Critical Care) with oxygen. To cause death in around 40–50% of CLP mice, 50% of the cecum was ligated and punctured twice with a 20-gauge needle. Saline (1 mL) was given subcutaneously for resuscitation immediately after operation. Mice were sacrificed at 18 h after CLP.Serum samples were collected for the detection of IL-1α, IL-1β, TNF-α, IL-6. alanine aminotransferase (ALT) and creatinine (Cre). Lung specimens were stained with H&E. To cause death in around 80% of CLP mice, 75% of the cecum was ligated and punctured twice with an 20-gauge needle. Mice were monitored daily by signs of a moribund state for lethality.

### Macrophages preparation and stimulation

Mouse peritoneal macrophages were isolated and cultured as described previously (Lu et al. 2012). Briefly, mice (7–12 wk. old) were intraperitoneally injected with 3 mL of sterile 4% thioglycollate broth to elicit peritoneal macrophages. Cells were collected by lavage of the peritoneal cavity with 5 mL of RPMI medium 1640 (Gibco)72 h later. After washing, cells were resuspended in RPMI medium 1640 (Gibco) supplemented with 10% heat-inactivated FBS and antibiotics (Gibco). Peritoneal macrophages (10^6^ cells per well) plated in 12-well plates were stimulated with LPS or CTB plus LPS. Supernatants were collected 16 h later for ELISA and LDH assay.

### Measuring ALT and creatinine

Serum samples were collected form indicated genotypes mice,ALT and Creatinine were measured by Automatic Biochemical Analyzer (Chemray240).

### ELISA and Cell death assay

Plasma and cell culture supernatant samples were analyzed using IL-1α (eBioscience), IL-1β (eBioscience), TNF-α (eBioscience), IL-6(eBioscience) ELISA kits. Cell death was assessed by LDH Cytotoxicity Assay kit (Beyotime Biotechnology).

### Isolation of cytosol fraction from mouse peritoneal macrophages and LPS activity assay

Subcellular fractions of mouse peritoneal macrophages were isolated by a digitonin-based fractionation method as described previously with modifications (Vanaja et al. 2016). Briefly, 5 × 10^6^ cells were stimulated with LPS (1 μg/ml) or CTB plus LPS (1 μg/ml) or CTB alone. After 2 h of treatment, the cells were washed with sterile cold PBS 4 times. Cells were subsequently treated with 300 μl of 0.005% digitonin extraction buffer for 20 min on ice and the supernatant containing cytosol was collected. The residual cell fractions containing cell membrane, organelles and nucleus were collected in 300 μl of 0.1% CHAPS buffer. BCA assay was used for protein quantification and LPS activity assay was used for LPS quantification. In addition, the fractions were subjected to immunoblot for Na+/K+ ATPase, Rab7, LAMP1, and beta-Actin to confirm the purity of cytosolic fraction.

### Statistical analysis

All data were analyzed using GraphPad Prism software (version 5.01). Data were analyzed by using two-tailed student’s t-test for comparison between two groups. Data were analyzed by using one-way ANOVA followed by post-hoc Bonferroni test for comparison between multiple groups. Survival data were analyzed using the log-rank test. A *p*-value < 0.05 was considered statistically significant for all experiments. All values are presented as the mean ± SD.

## Results

### TRIF is required for caspase-11-dependent immune responses in endotoxemia

**Fig. 1:**
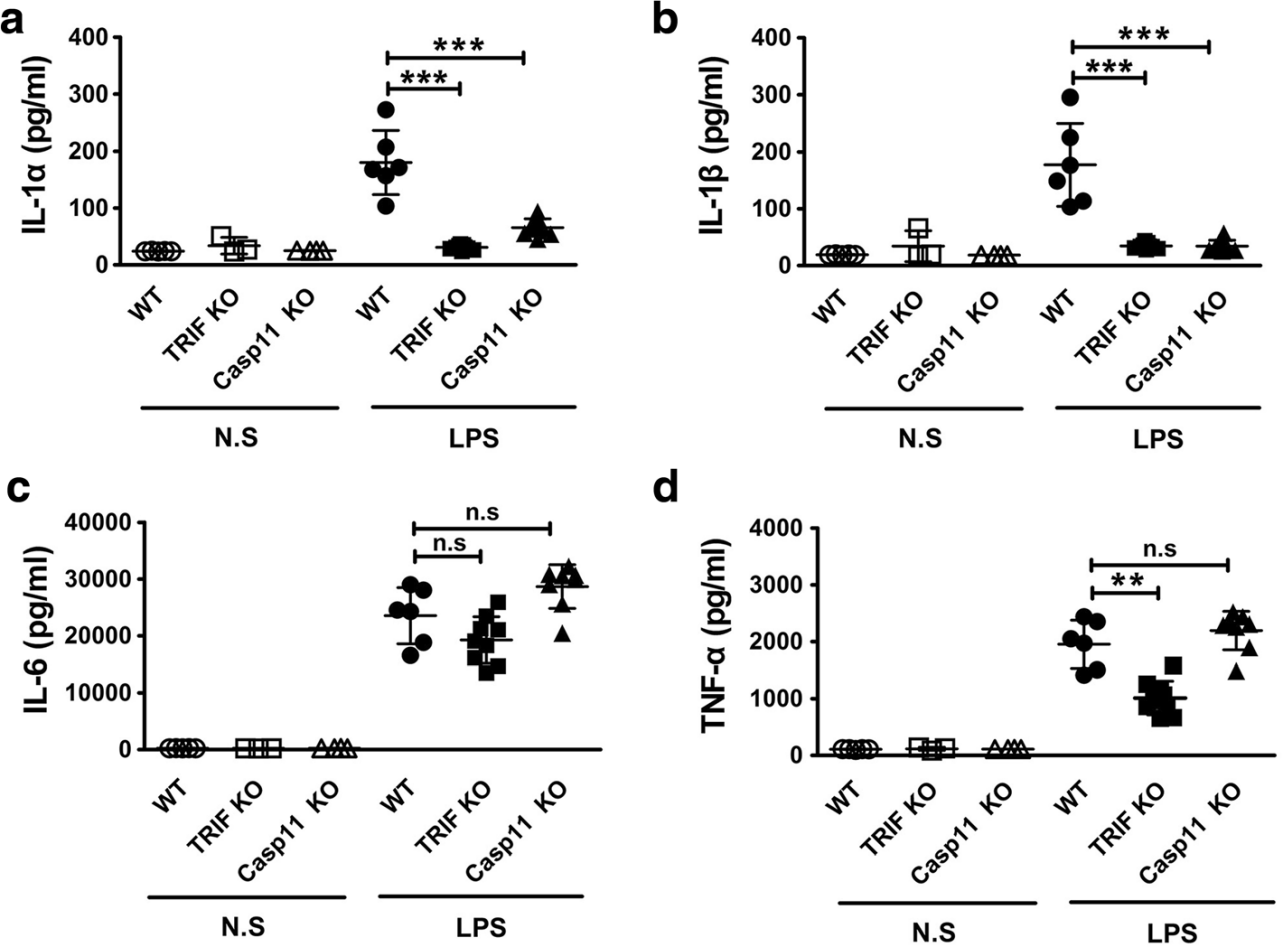
TRIF is required for caspase-11-dependent immune responses in endotoxemia. Serum IL-1α (**a**), IL-1β (**b**), TNF-α (**c**) and IL-6 (**d**) levels from mice of indicated genotypes following intraperitoneal injection with either 10 mg/kg LPS or saline. Serum samples were collected 16 h after LPS injection. *P<0.05; **P<0.01; ***P<0.001 (Student’s t-test). Graphs show the mean ± SD. Data are representative of at least three independent experiments

To determine whether TRIF is critical for caspase-11-dependent immune responses in endotoxemia, wild-type (WT), TRIF KO and Caspase-11 KO mice were used. Consistent with previous works (Kayagaki et al. 2011; Hagar et al. 2013; Kayagaki et al. 2013; Wang et al. 1998; Cheng et al. 2017), genetic deletion of Caspase-11 blocked the release of IL-1α and IL-1β in endotoxemia (Fig. 1a-b). Notably, TRIF deficiency abolished endotoxemia-induced IL-1α and IL-1β release (Fig. 1a-b). In contrast, deletion of TRIF failed to inhibit IL-6 release (Fig. 1c), which does not rely on caspase-11 or pyroptosis. Previous studies establish that TRIF signaling is important for tumor necrosis factor (TNF) production (Gais et al. 2010). In agreement with these works, we observed that serum levels of TNF in TRIF KO mice were significantly lower than those of in WT mice (Fig. 1d). Taken together, these findings indicate that TRIF is critical for caspase-11-dependent immune responses in endotoxemia.

### TRIF is required for caspase-11-dependent organ injury and lethality in endotoxemia

**Fig. 2:**
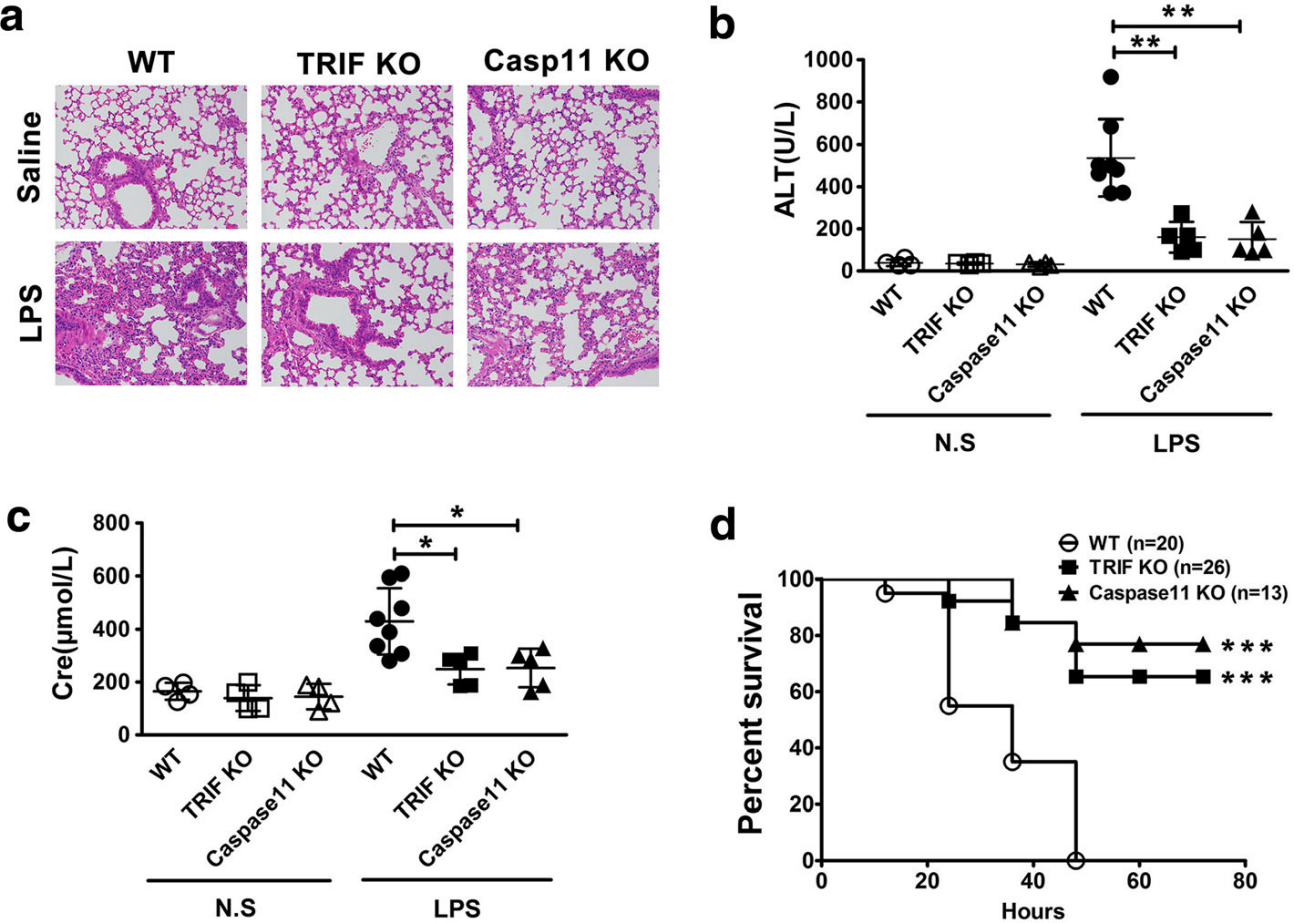
TRIF is required for caspase-11-dependent organ injury and lethality in endotoxemia. **a** Histology of the lungs from mice of indicated genotypes (*n* = 5) following intraperitoneal injection with either 10 mg/kg LPS or saline. Lungs were collected 8 h after LPS injection and then stained with H&E. **b-c** The serum alanine aminotransferase (ALT) and creatinine (Cre) levels from mice of indicated genotypes following intraperitoneal injection with either 10 mg/kg LPS or saline. Serum samples were collected 8 h after LPS injection. **d** Kaplan Meier survival curves for the indicated genotypes mouse strains subjected to 40 mg/kg LPS injection. *P<0.05; **P<0.01; ***P<0.001 (Student’s t-test and log-rank test for survival). Graphs show the mean ± SD. Data are representative of at least three independent experiments

Endotoxemia-induced lung injury and lethality largely depend on caspase-11 (Kayagaki et al. 2011; Cheng et al. 2017). Next we determine whether TRIF is required for caspase-11-dependent lung injury and lethality in endotoxemia. Consistent with previous report, deletion of Caspase-11 prevented endotoxemia-induced pulmonary leukocyte infiltration (Fig. 2a). Notably, TRIF deficiency also blocked pulmonary leukocyte infiltration after LPS challenge (Fig. 2a). Next we measured serum levels of alanine aminotransferase (ALT), and creatinine (Cre) to determine whether TRIF is required for caspase-11-dependent liver and kidney injury. Deletion of TRIF or Caspase-11 markedly attenuated the elevation of the serum levels of ALT and Cre in endotoxemia (Fig. 2b-c). Further, Deletion of TRIF or Caspase-11 prevented lethality in endotoxemia (Fig. 2d). Thus, TRIF is required for caspase-11-dependent organ injury and lethality in endotoxemia.

### TRIF is critical for caspase-11-dependent immune responses, organ injury and lethality in sepsis

**Fig. 3:**
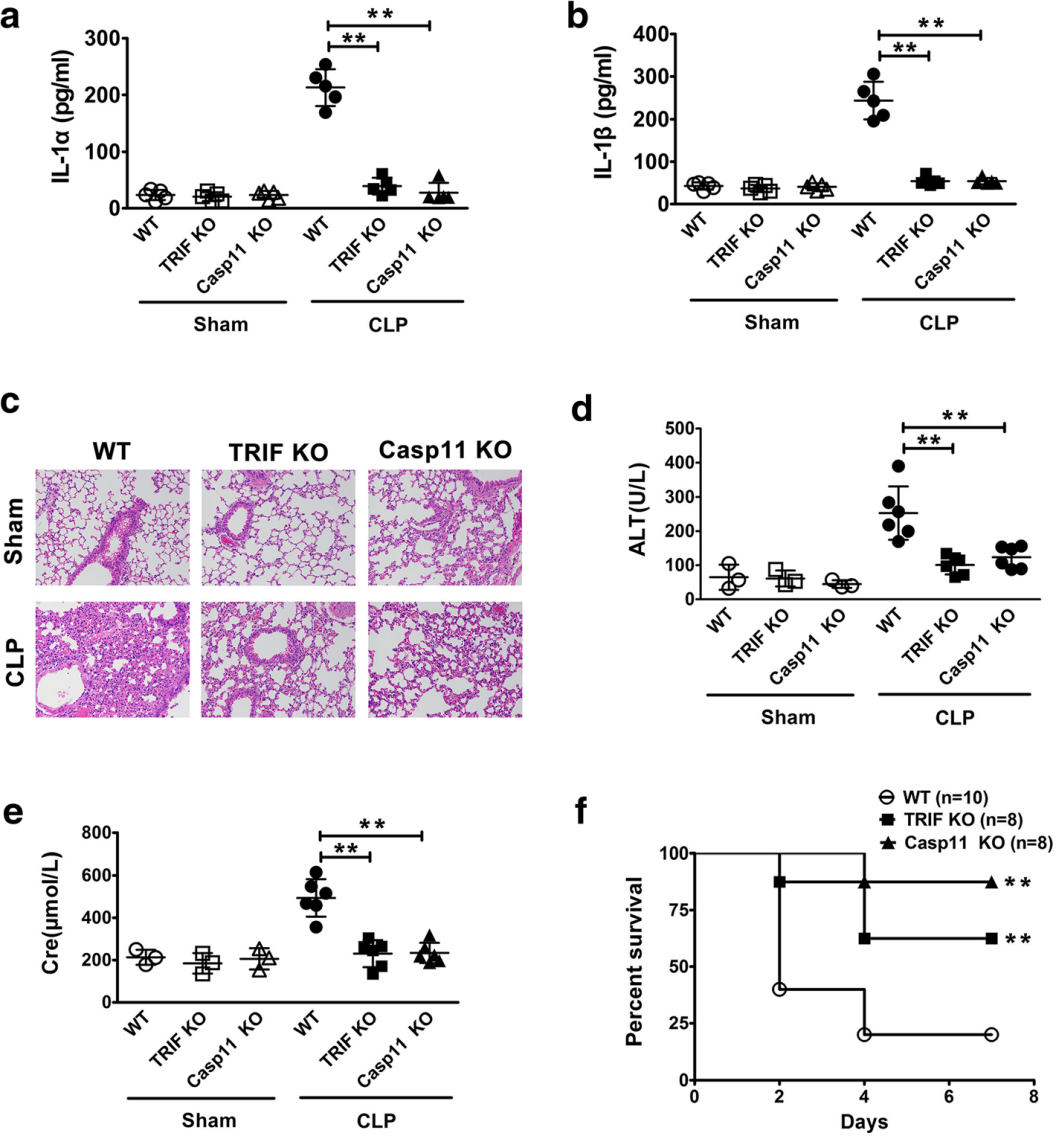
TRIF is critical for caspase-11-dependent immune responses, organ injury and lethality in sepsis. **a-b** Serum IL-1α and IL-1β levels from mice of indicated genotypes were subjected to either cecum sublethal ligation and puncture (CLP) or sham operation. **c** H&E staining of the lungs from mice of indicated genotypes (*n* = 5) were subjected to either sublethal CLP or sham operation. **d-e** The serum alanine aminotransferase (ALT) and creatinine (Cre) levels from mice of indicated genotypes subjected to either sublethal CLP or sham operation. **f** Kaplan Meier survival curves for the indicated genotypes subjected to either sublethal CLP or sham operation. *P<0.05; **P<0.01; ***P<0.001 (Student’s t-test and log-rank test for survival). Graphs show the mean ± SD. Data are representative of at least three independent experiments

Experiments were then conducted to determine whether TRIF is required for caspase-11 activation in bacterial sepsis. Using cecum ligation and puncture (CLP), a clinically relevant murine model of Gram-negative polymicrobial sepsis, we observed that deletion of TRIF or Caspase-11 blocked the release of IL-1α and IL-1β (Fig. 3a-b), the pulmonary leukocyte infiltration (Fig. 3c), the elevation of the serum levels of ALT and Cre (Fig. 3d-e), and the lethality (Fig. 3f). Together, these findings demonstrated that TRIF is critical for caspase-11-dependent immune responses, organ injury and lethality in bacterial sepsis.

### Type 1 interferon signaling is critical for caspase-11-dependent immune responses, organ injury and lethality in endotoxemia

**Fig. 4:**
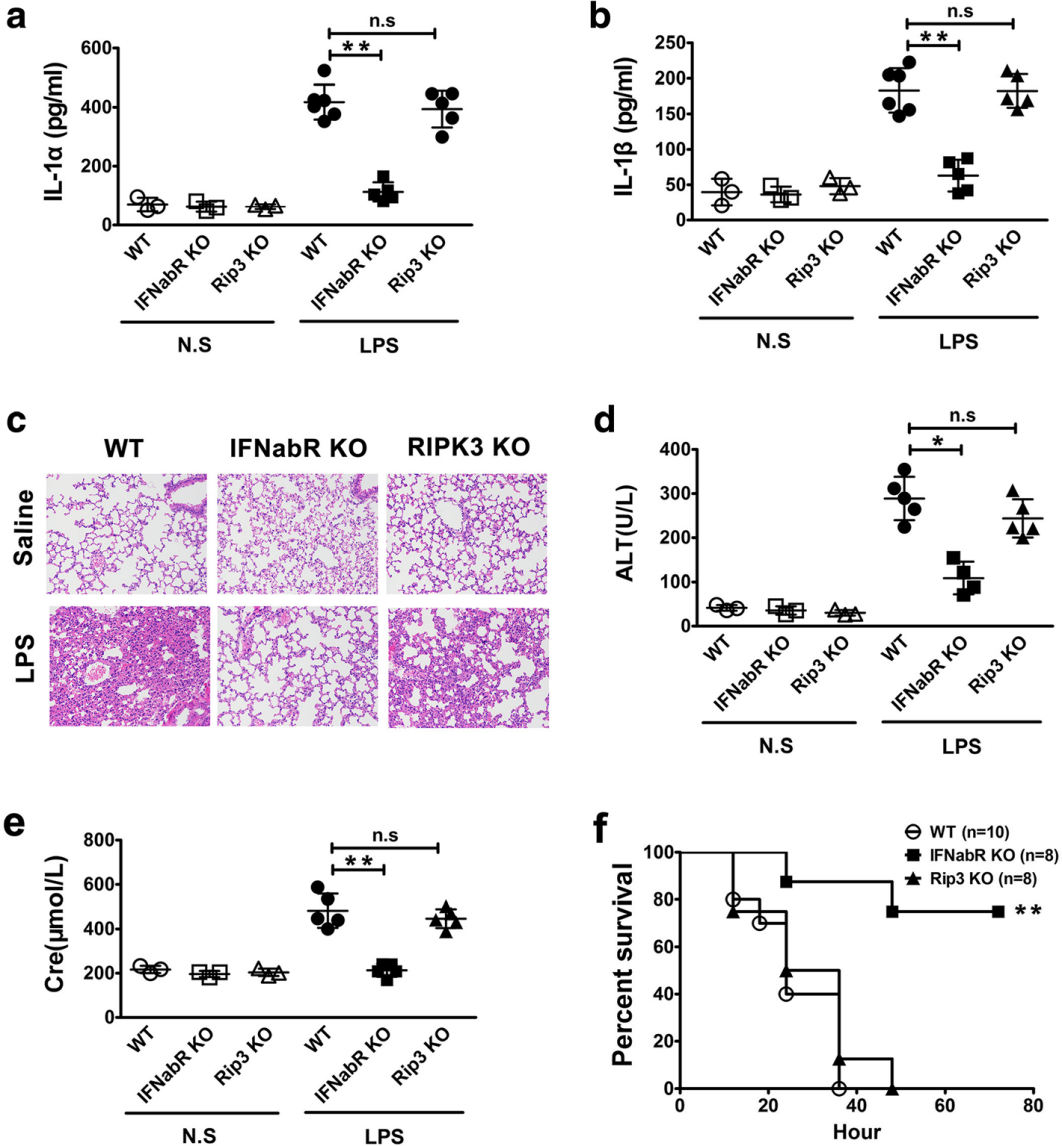
Type 1 interferon signaling is critical for caspase-11-dependent immune responses, organ injury and lethality in endotoxemia. **a-b** Serum IL-1α and IL-1β levels from mice of indicated genotypes following intraperitoneal injection with either 10 mg/kg LPS or saline. **c** H&E staining of the lungs from mice of indicated genotypes (*n* = 5) following intraperitoneal injection with either 10 mg/kg LPS or saline. **d-e** The serum alanine aminotransferase (ALT) and creatinine (Cre) levels from mice of indicated genotypes following intraperitoneal injection with either 10 mg/kg LPS or saline. **f** Kaplan Meier survival curves for the indicated genotypes following intraperitoneal injection with 40 mg/kg LPS. *P<0.05; **P<0.01; ***P<0.001 (Student’s t-test and log-rank test for survival). Graphs show the mean ± SD. Data are representative of at least three independent experiments

Upon TLR4 or TLR3 activation, TRIF triggers the production of type 1 interferon through recruitment of its downstream factor interferon regulatory factor 3 (IRF3) (Yamamoto et al. 2003). TRIF signaling also induces the activation of receptor interacting protein kinase 3 (Rip3), which is a serine/threonine kinase crucial for programmed necrosis (also termed necroptosis) (He et al. 2011). Thus, we next examined whether type 1 IFN signaling or Rip3 is critical for endotoxemia-induced caspase-11 activation. Deletion of IFN-α/βR, the receptor for type 1 IFN, but not Rip3, significantly inhibited the release of IL-1α and IL-1β in endotoxemia (Fig. 4a-b). Moreover, IFN-α/βR deficiency blocked the pulmonary leukocyte infiltration (Fig. 4c), the elevation of the serum levels of ALT and Cre (Fig. 4d-e), and the lethality in endotoxemia (Fig. 4f). These observations establish that type 1 interferon signaling is critical for caspase-11-dependent immune responses, organ injury and lethality in endotoxemia.

### GBPs are essential for caspase-11-dependent immune responses and lethality in endotoxemia

**Fig. 5:**
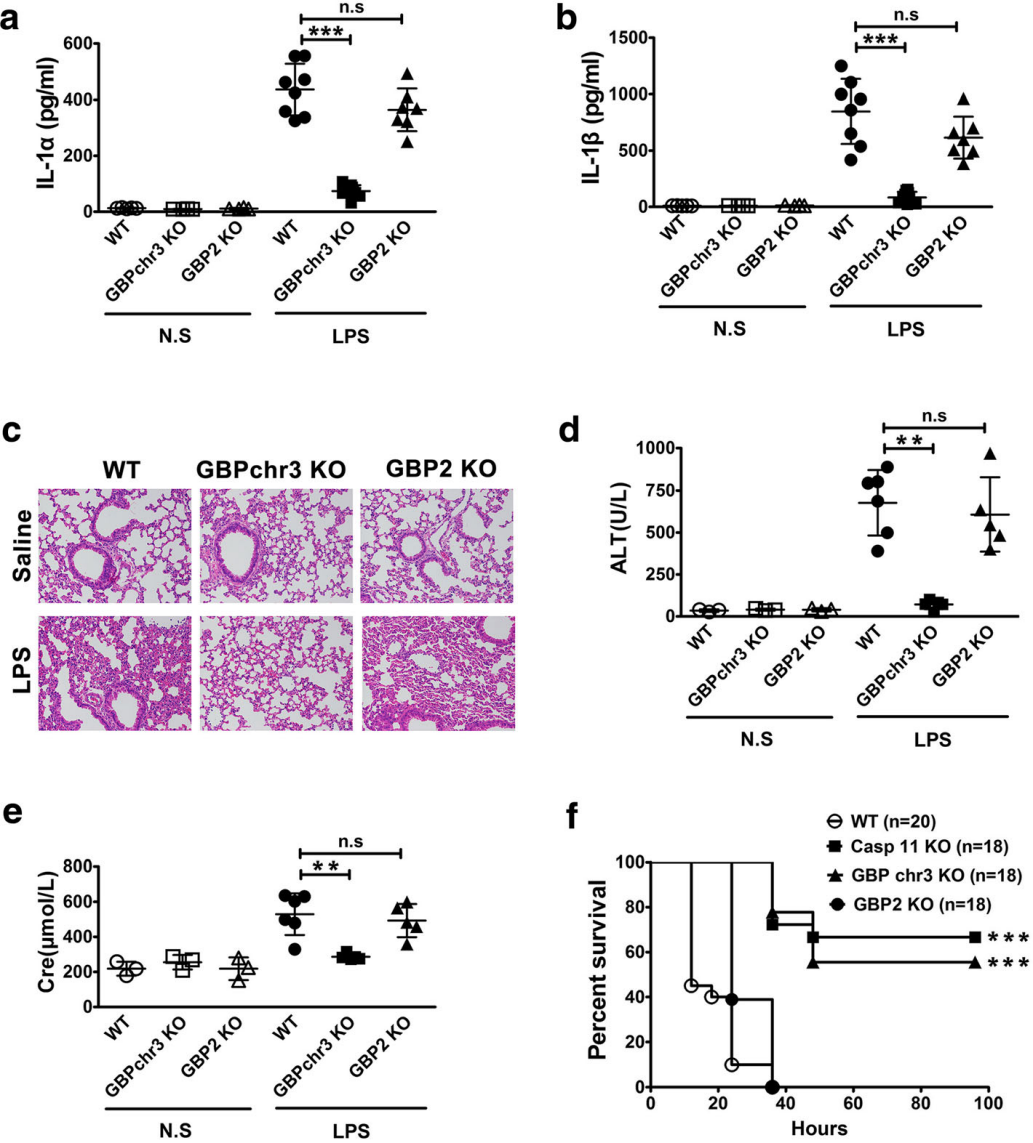
GBPs are essential for caspase-11-dependent immune responses and lethality in endotoxemia. **a-b** Serum IL-1α and IL-1β levels from mice of indicated genotypes following intraperitoneal injection with either 10 mg/kg LPS or saline. **c** H&E staining of the lungs from mice of indicated genotypes (*n* = 5) following intraperitoneal injection with either 10 mg/kg LPS or saline. **d-e** The serum alanine aminotransferase (ALT) and creatinine (Cre) levels from mice of indicated genotypes following intraperitoneal injection with either 10 mg/kg LPS or saline. **f** Kaplan Meier survival curves for the indicated genotypes following intraperitoneal injection with 40 mg/kg LPS .*P<0.05; **P<0.01; ***P<0.001 (Student’s t-test and log-rank test for survival). Graphs show the mean ± SD. Data are representative of at least three independent experiments

Type 1 IFNs stimulation leads to expression of hundreds of genes, termed IFN-stimulated genes (ISGs), in both immune and non-immune cells. Among the most strongly upregulated ISGs were interferon induced GTPases, such as guanylate-binding proteins (GBPs) (Meunier et al. 2014; Finethy et al. 2017; Santos et al. 2018; Gu et al. 2018). Previous works show that GBPs are critical for Gram-negative bacteria-induced caspase-11 activation, with GBP2 playing the dominant role in this scenario (Meunier et al. 2014). A recent study reported that GBP2 is required for OMVs-induced caspase-11 activation in IFN-γ-primed mouse macrophages (Finethy et al. 2017). However, deletion of GBP2 only slightly inhibited the release of IL-1α and IL-1β in endotoxemia (Fig. 5a-b). Accordingly, GBP2 deficiency failed to significantly reduced lethality in endotoxemia (Fig. 5f). These findings implicate the redundant roles of GBP family proteins in mediating caspase-11 activation in endotoxemia.

To test this end, we utilized the GBP chr3 KO mice, in which GBP1, GBP2, GBP3, GBP5 and GBP7 have been deleted (Meunier et al. 2014). Notably, deletion of GBPs markedly reduced the release of IL-1α and IL-1β in endotoxemia (Fig. 5a-b). In accordance, GBPs deficiency blocked pulmonary leukocyte infiltration (Fig. 5c), elevation of the serum levels of ALT and Cre (Fig. 5d-e), and lethality in endotoxemia (Fig. 5f). Together, GBP family proteins play redundant roles in mediating caspase-11 activation in endotoxemia.

### Type 1 IFNs-GBPs pathway is critical for caspase-11-dependent immune responses, organ injury and lethality in sepsis

**Fig. 6:**
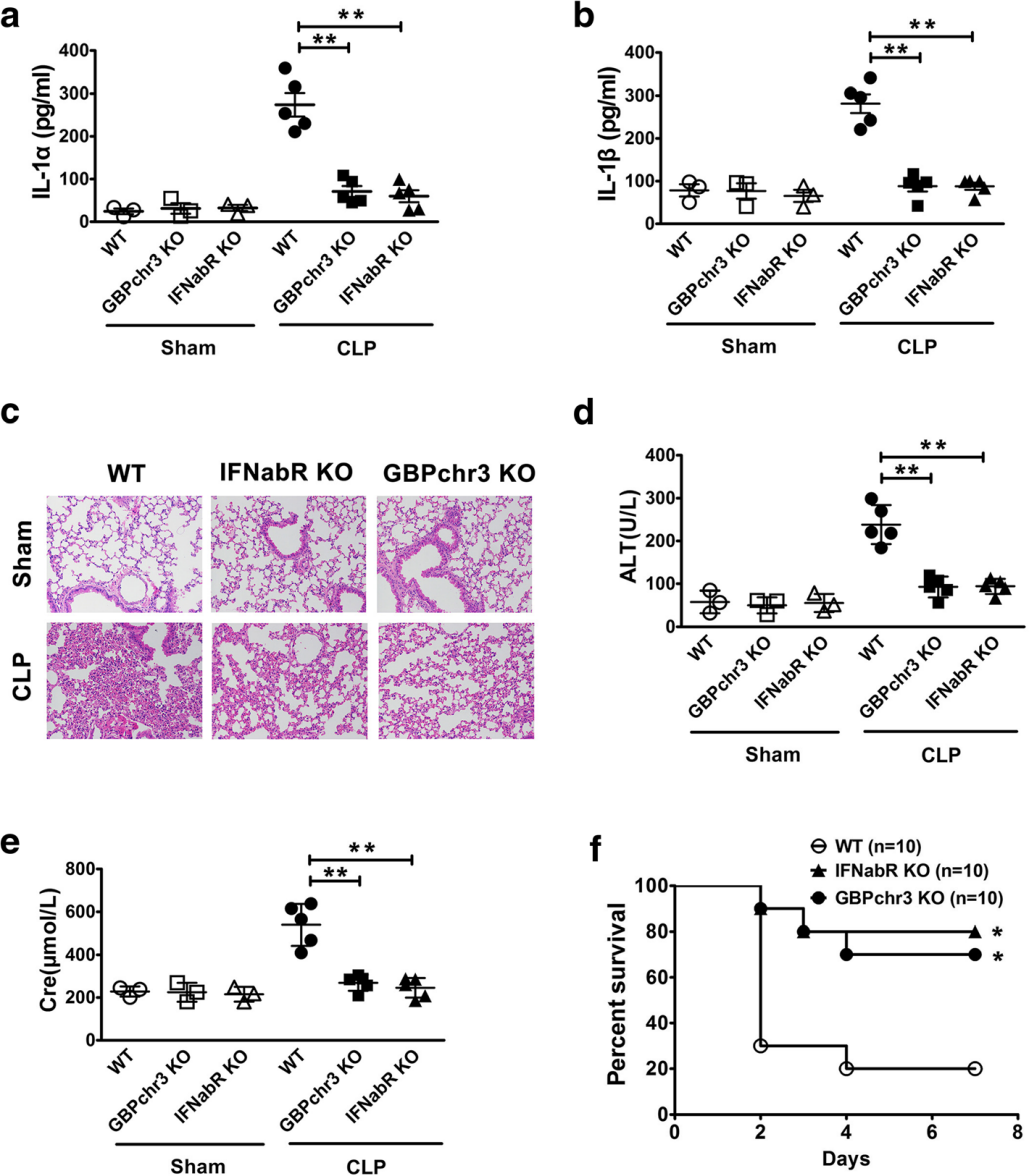
Type 1 IFNs-GBPs pathway is critical for caspase-11-dependent immune responses, organ injury and lethality in sepsis. **a-b** Serum IL-1α and IL-1β levels from mice of indicated genotypes subjected to either CLP or sham operation. **c** H&E staining of the lungs from mice of indicated genotypes (*n* = 5) subjected to either CLP or sham operation. **d-e** The serum alanine aminotransferase (ALT) and creatinine (Cre) levels from mice of indicated genotypes were subjected to either CLP or sham operation. **f** Kaplan Meier survival curves for the indicated genotypes subjected to either CLP or sham operation.*P<0.05; **P<0.01; ***P<0.001 (Student’s t-test and log-rank test for survival). Graphs show the mean ± SD. Data are representative of at least three independent experiments

Next we determined whether type 1 IFNs-GBPs pathway is critical for caspase-11-dependent immune responses and lethality in bacterial sepsis. Using CLP models, we observed that deletion of IFN-α/βR or GBPs blocked the release of IL-1α and IL-1β (Fig. 6a-b), the pulmonary leukocyte infiltration (Fig. 6c), the elevation of the serum levels of ALT and Cre (Fig. 6d-e), and the lethality (Fig. 6f), all of which depends on caspase-11 (Fig. 3f). Thus, these findings indicated that type 1 IFNs-GBPs pathway is critical for caspase-11-dependent immune responses, organ injury and lethality in bacterial sepsis.

### TRIF-interferon-GBPs pathway is required for caspase-11 activation in vitro

**Fig. 7:**
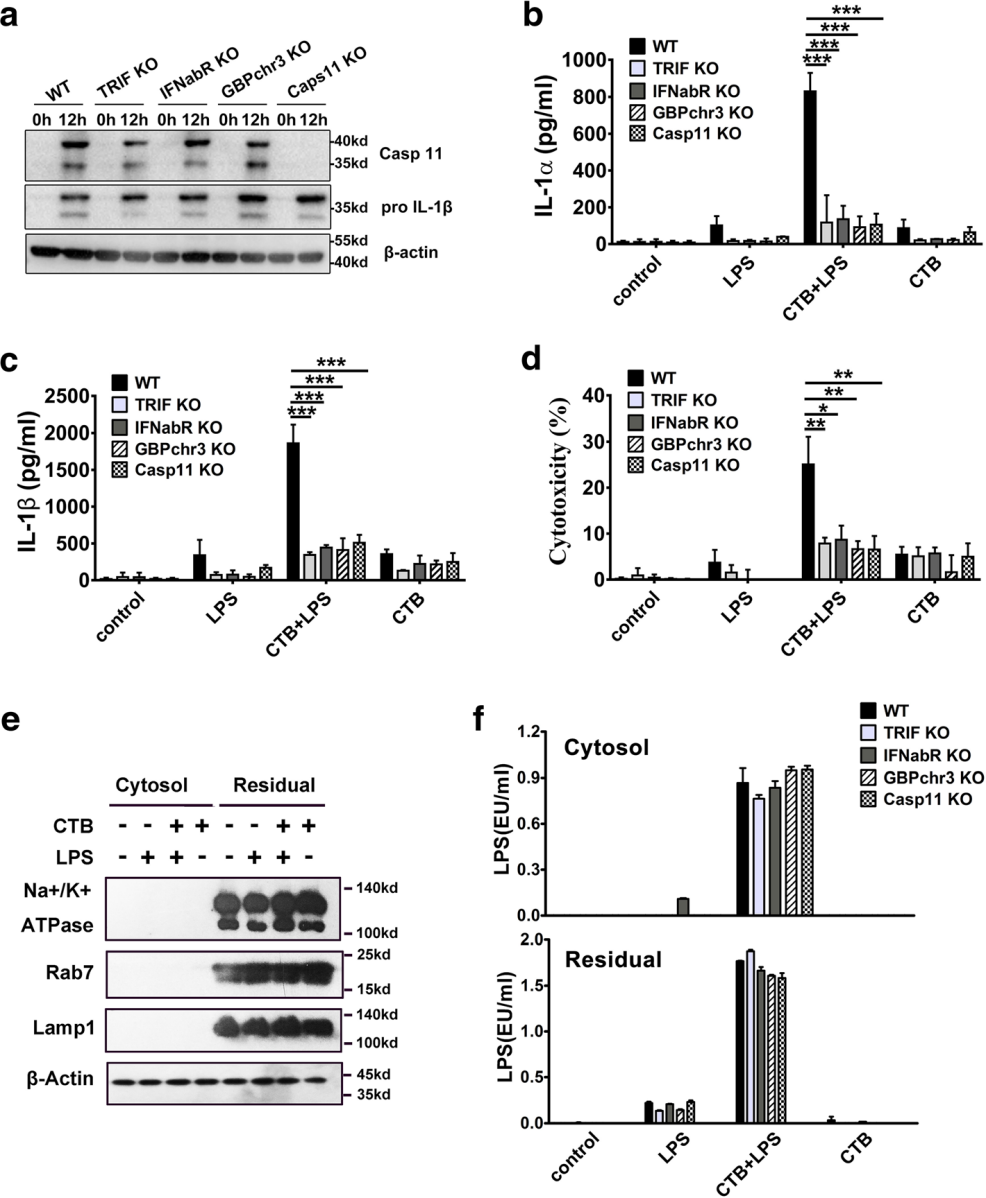
TRIF-interferon-GBPs pathway is required for caspase-11 activation in vitro. **a** Western-blot to detect the expression of caspase-11, IL-1β and β-Actin in splenocytes isolated from mice of indicated genotypes following intraperitoneal injection with either 10 mg/kg LPS or saline. **b-d** ELISA for IL-1α, IL-1β and LDH assay in the supernatants of peritoneal macrophages stimulated with either LPS (1 μg/ml) alone or LPS (1 μg/ml) plus CTB. Supernatants were collected 16 h after stimulation. **e** Western-blot for Na+/K+ ATPase, Rab7, LAMP1 and β-actin in the cytosolic and residual fractions from LPS (1 μg/ml) or CTB plus LPS (1 μg/ml) or CTB alone -stimulated mouse peritoneal macrophages obtained by digitonin fractionation. **f** LPS activity assay in the cytosolic and residual fractions from LPS (1 μg/ml) or CTB plus LPS (1 μg/ml) or CTB alone -stimulated mouse peritoneal macrophages obtained by digitonin fractionation.*P<0.05; **P<0.01; ***P<0.001 (Student’s t-test). Graphs show the mean ± SEM. Data are representative of at least three independent experiments

To determine whether TRIF-interferon pathway is essential for caspase-11 expression, WT, TRIF KO, IFNabR KO, GBPchr3 KO and Caspase-11 KO mice were subjected to lethal endotoxemia. As revealed by western-blot, the expression of caspase-11, IL-1β and β-Actin in the spleens were comparable among WT, TRIF KO, IFNabR KO and GBPchr3 KO mice (Fig. 7a). These observations indicate that TRIF-type 1 IFNs pathway is critical for the activation rather than the expression of caspase-11 and IL-1β.

We next investigated how TRIF-interferon-GBPs pathway regulates caspase-11 activation*.* Extracellular LPS is able to induce caspase-11-dependent pyroptosis of cultured mouse macrophages in the presence of cholera toxin B^3^. Using this in vitro caspase-11 activation model, we found that TRIF, type 1 IFNs and GBPs are required for caspase-11-dependent release of IL-1α, IL-1β and LDH (Fig. 7b-d). To exclude the possibility that TRIF-interferon-GBPs pathway is essential for the translocation of extracellular LPS to the cytosol, we isolated cytosol devoid of cytoplasmic membranes, endosomes and lysosomes using low concentrations of digitonin on mouse macrophages treated with LPS and cholera toxin B (Fig. 7e). LPS levels in the cytosolic fraction were comparable among WT, TRIF KO, IFN-α/βR KO, or GBPchr 3 KO macrophages (Fig. 7f). As TRIF-type 1 IFN signaling is essential for the expression of GBPs, these data indicate that GBPs are critical for cytosolic LPS-induced activation.

## Discussion

Previous studies show that TRIF signaling and GBPs are critical for vacuolar Gram-negative bacteria-induced caspase-11 activation (Meunier et al. 2014; Rathinam et al. 2012). In this context, vacuolar Gram-negative bacteria induce the expression of GBPs, which target the bacteria-containing vacuoles and subsequently induce lysosomal rupture (Meunier et al. 2014). This event results in the leakage of LPS into the cytoplasm, leading to caspase-11 activation (Meunier et al. 2014). However, recent advance reveals that extracellular Gram-negative bacteria are also capable of activating caspase-11 in adjacent macrophages (Vanaja et al. 2016). In this regards, phagocytosis of the whole bacteria and lysosomal rupture are not necessary for the activation of caspase-11 (Vanaja et al. 2016). One of the underlying mechanisms is that Gram-negative bacteria-released OMVs deliver LPS into the cytoplasm (Vanaja et al. 2016). Another mechanism through which Gram-negative bacterial infection triggers caspase-11-dependent immune responses is endotoxemia. Endotoxemia is common in sepsis; and removal of circulating LPS is beneficial to septic patients (Angus and van der Poll 2013; Ronco et al. 2010). Importantly, endotoxemia is able to trigger robust caspase-11-dependent immune responses in a manner similar to Gram-negative bacteremia (Kayagaki et al. 2011; Hagar et al. 2013; Kayagaki et al. 2013; Wang et al. 1998; Cheng et al. 2017). However, the upstream pathways that regulate endotoxemia-induced caspase-11 activation remain largely unknown. In current study, we showed for the first time that TRIF-type 1 IFN-GBPs signaling is critical for caspase-11-dependent immune responses, organ injury and lethality in both endotoxemia and polymicrobial sepsis.

Among GBP protein family, GBP2 plays the dominant role in vacuolar Gram-negative bacteria-induced caspase-11 activation (Meunier et al. 2014). Interestingly, we found that GBP2 deficiency fails to significantly inhibit caspase-11-dependent immune responses in endotoxemia; whereas genetic deletion of GBP1, GBP2, GBP3, GBP5 and GBP7 simultaneously blocked endotoxemia-induced caspase-11 activation. These observations suggest that GBP proteins play redundant and distinct roles in mediating caspase-11 activation in responses to different stimuli. The mechanisms by which GBPs mediate caspase-11 activation are not fully understood. Early work shows that GBPs enable LPS leaking into the cytosol by inducing lysosomal rupture (Meunier et al. 2014). However, accumulated evidence reveals that GBPs are also essential for intracellular LPS-induced caspase-11 activation (Santos et al. 2018; Pilla et al. 2014). In line with these findings, we found that GBPs are required for the caspase-11 activation but not the cytoplasmic translocation of LPS in cholera toxin B + LPS-stimulated macrophages. As GBPs could physically bind LPS, one intriguing possibility is that GBPs might function as a co-receptor of intracellular LPS for caspase-11. Taken together, our study identifies the TRIF-type 1 IFN-GBPs signaling as an upstream pathway that mediates caspase-11 activation, and suggests that targeting this pathway might be potential therapeutic strategy to treat sepsis.

## Conclusions

Together, our findings demonstrate that TRIF signaling is required for caspase-11-dependent immune responses and lethality in endotoxemia and sepsis, and provide novel mechanistic insights into how LPS induces caspase-11 activation during bacterial infection.

## Abbreviations

ALT: Alanine aminotransferase
CLP: Cecum ligation and puncture
Cre: Creatinine
GBPs: Guanylate-binding proteins
GSDMD: Gasdermin D
IL-1α: Interleukin-1α
IL-1β: Interleukin-1β
IL-6: Interleukin-6
LPS: Lipopolysaccharide
NLRP3: NLR pyrin domain-containing protein 3
OMVs: Bacterial outer membrane vesicles
oxPAPC: Oxidized phospholipid
Rip3: Receptor interacting protein 3
TALENs: Transcription activator-like effector nucleases
TNF-α: Tumor necrosis factor-α
TRIF: TIR-domain-containing adapter-inducing interferon-β
